# Microtubule cytoskeleton and mycorrhizal roots

**DOI:** 10.1080/15592324.2022.2031504

**Published:** 2022-02-01

**Authors:** Tania Ho-Plágaro, María Isabel Tamayo-Navarrete, José M. García Garrido

**Affiliations:** Department of Soil Microbiology and Symbiotic Systems, Estación Experimental del Zaidín (EEZ), CSIC, Granada, Spain

**Keywords:** Arbuscular mycorrhiza, microtubule, cytoskeleton

## Abstract

For the establishment of the Arbuscular Mycorrhiza (AM) symbiosis it is essential that epidermis and cortical cells from plant roots suffer a strong reorganization to allow the penetration of intracellular fungal hyphae. In the same manner, the new formation of a periarbuscular membrane and a symbiotic interface with specific compositions are required for a functional symbiosis. It is believed that the cytoskeleton of the plant host plays an essential role in these processes, particularly the microtubule (MT) cytoskeleton, as huge modifications have been observed in the MT array of root cells accompanying the establishment of the AM symbiosis. Recent research has established a link between microtubule rearrangements and arbuscule functioning. However, further research is required to elucidate the specific functions of MT cytoskeleton along the different stages of the arbuscule life cycle and to unravel the signals triggering these changes.

For the establishment of the Arbuscular Mycorrhiza (AM) symbiosis it is essential that epidermis and cortical cells from plant roots suffer a strong reorganization to allow the penetration of intracellular fungal hyphae. In the same manner, the new formation of a periarbuscular membrane and a symbiotic interface with specific compositions are required for a functional symbiosis. It is believed that the cytoskeleton of the plant host plays an essential role in these processes, particularly the microtubule (MT) cytoskeleton, as huge modifications have been observed in the MT array of root cells accompanying the establishment of the AM symbiosis. Recent research^1^ has established a link between microtubule rearrangements and arbuscule functioning. However, further research is required to elucidate the specific functions of MT cytoskeleton along the different stages of the arbuscule life cycle and to unravel the signals triggering these changes.

## Microtubule cytoskeleton alterations in mycorrhizal roots

Numerous evidences exist showing changes of the MT cytoskeleton in mycorrhizal roots. From the first contact of the fungal hypha with the root surface, it has been observed that the contacted epidermal cell suffers rearrangements in their cortical microtubules, from an oblique organization to a random array.^2^ When AM fungi colonize the root, similar changes have been observed in arbuscule-hosting cells at the early stages of arbuscule development and also in non-colonized cortical cells adjacent to arbuscule-containing cells.^3^

Along the formation of the arbuscules, the original cortical MTs are progressively fragmented and finally disappear, while other new MTs bundles, surrounding the arbuscule and connecting the arbuscular hypha among them and with the cell nucleus, are formed.^3–5^ Later, in completely mature arbuscules the MT fibers are found to get thinner and more fragmented.^3^ Finally, in senescent arbuscules, new long cortical MTs bundles transiently appear, together with the synthesis of the new helicoidal array typically found in root cortex cells.^3^

Modifications have also been observed regarding the MT organizing center (MTOC), which controls the MT assembling and organization. While the MTOC is usually located surrounding the cell nucleus, in cells colonized by AM fungi the MTOC (visualized through γ-tubulin labeling) changes to a diffuse array surrounding the fine branches of the arbuscule.^6^

It is also worth mentioning that in the cells colonized by AM fungi, it seems that the tubulin synthesis is increased. For example, the expression of the mays gene *Tubα3*, which encodes a tubulin, is induced in the arbuscule-hosting cells.^7^ In addition, through fluorescent labeling, it has been observed that the amount of α – tubulin (which corresponds to the MTs) and γ – tubulin (corresponding to the MTOC) is increased in AM colonized cells.^5,6^

## The role of MT rearrangements in mycorrhizal roots

MTs are well-known to play a role in determining cell shape and organelle transport.^8,9^ They also act as tracks for intracellular clathrin-coated vesicle trafficking (CCVs), the major cellular means of transport, and direct exocytosis and endocytosis pathways.^10,11^ In this manner, the MT cytoskeleton has an essential role in the establishment of cell polarity, cell wall formation, and plasma membrane expansion, differentiation and recycling.^12–14^ All these processes occur in plant root cells hosting AM arbuscules and are required for a functional symbiosis. In these cells, the establishment of a specialized cell wall and membrane surrounding the arbuscule is crucial for mycorrhizal development and functioning.^15^ Polarized and asymmetric delivery of cell wall components^16,17^ and intrinsic membrane proteins^18,19^ is therefore required. Actually, arbuscule development has been described to be accompanied by specific vesicle trafficking.^20–23^ Although substantial cytoskeletal remodeling has been reported in arbuscule-containing cells,^3,6^ it is not known if they are involved in all these processes and research being done is very scarce.

To our knowledge, the first investigation regarding the role of MTs during mycorrhization^24^ reported that disruption of microtubules by oryzalin in tomato AM infected roots did not significantly affect the percentage of AM colonization. However, they didn´t check if arbuscule morphology or arbuscule functionality were altered. As previously suggested,^25^ the study of MT-associated proteins (MAPs) might be the best strategy to understand the relationship between MT cytoskeletal remodeling and mycorrhizal development and/or function. To date, only a recent research has deepened into this question.^1^ In this study, the authors identified the tomato *Tsb* gene induced in arbuscule-hosting cells and encoding a putative MAP. Their results pointed to a role of this protein for MT bundling and for proper arbuscule functionality. Moreover, they suggest that MT rearrangements are also necessary at later stages of arbuscule life cycle, when the unbundling of microtubules might be required for the correct turnover of senescent arbuscules.

*Tsb* gene has previously been described as a gene exclusively expressed in pollen.^26^ TSB-like proteins seem to constitute a Solanaceae family-specific group of proteins. Gene structure (three exons and two introns), protein motifs related to MT binding (repeat motifs V-V-K-K-N/E-E), and the presence of many potential casein kinase II phosphorylation sites were common features conserved in all TSB-like proteins. Members of these group have been previously functionally characterized and all of them have been reported to function as MAPs able to bind and bundle MTs and to be involved in cytoskeletal remodeling during pollen development.^27–29^

## A biological machinery for MT rearrangements commonly shared in mycorrhizal root colonization and pollen tube development?

The presence of a specific biological machinery commonly shared by arbuscule-containing cells and pollen tube cells has been suggested,^30^ probably due to the fact that both cells types are subjected to strong polar modifications. Actually, transcriptomic comparisons performed in *Medicago* and tomato revealed significant overlaps between arbuscular mycorrhiza and pollen development,^1,30^ with a predominance of genes related to the creation and modification of membranes and cell-walls. One of these overlapping genes is the tomato *Tsb* gene, which is specifically induced in pollen and in mycorrhizal roots.^1,26^ Moreover, the study concerning the *Tsb* gene,^1^ together with research related to several *Tsb*-Solanaceae homologs,^27–29^ suggests that the TSB protein is probably a MAP able to bind and bundle microtubules, with an essential role in the cytoskeleton remodeling during both processes, i.e. arbuscule formation and pollen development. These findings point to a possible common evolutionary origin of cytoskeleton-related mechanisms in the formation of arbuscules and pollen tubes. Then, the screening of pollen tube-related MAPs and their functional characterization in the mycorrhizal symbiosis may help to identify key mechanisms underlying MT modifications in mycorrhizal roots, and to understand the role of these MT rearrangements in the symbiosis.

## Signals that trigger the cytoskeleton changes during mycorrhization

The particular signals responsible for the MT cytoskeleton changes during mycorrhization are unknown. A mechanical stimulus exerted by the contact of the AM fungus with the plant cell should not be excluded as a signal for MT rearrangements, as previously suggested.^31^ However, to achieve the specific modifications occurring in root cells colonized by AM fungi, it is expected that this mechanical stimulus must be combined with a series of chemical signals. Supporting this idea, it has been reported that also MTs from non-colonized cortical cells adjacent to arbuscule-containing cells changed their structure,^3^ what strongly suggests that cortical cells are able to initiate MT cytoskeleton modifications prior to the entry of the fungus through a chemical signaling.

A complex hormonal regulation occurs in mycorrhizal roots,^32–34^ including hormones such as auxin, gibberellin, ethylene and abscisic acid, that have been reported to be involved in microtubule rearrangements in other cell types.^12,35–37^ Curiously, strigolactones, a plant hormone with crucial roles in pre-symbiotic chemical dialog between AM fungi and plant roots and necessary for the punctual entry of the fungus,^38–40^ have been also recently described to affect the organization of cortical microtubules.^41^ Additionally, coumarins, which have emerged as novel metabolites capable of stimulating AM fungi in the pre-penetration phase,^42^ have also been related with microtubule cortical array organization.^43^ However, the particular hormonal signaling or other kind of mechanisms involved in the regulation of cytoskeleton changes in AM colonized cells remain to be elucidated.

## Future challenges

**Figure 1.**
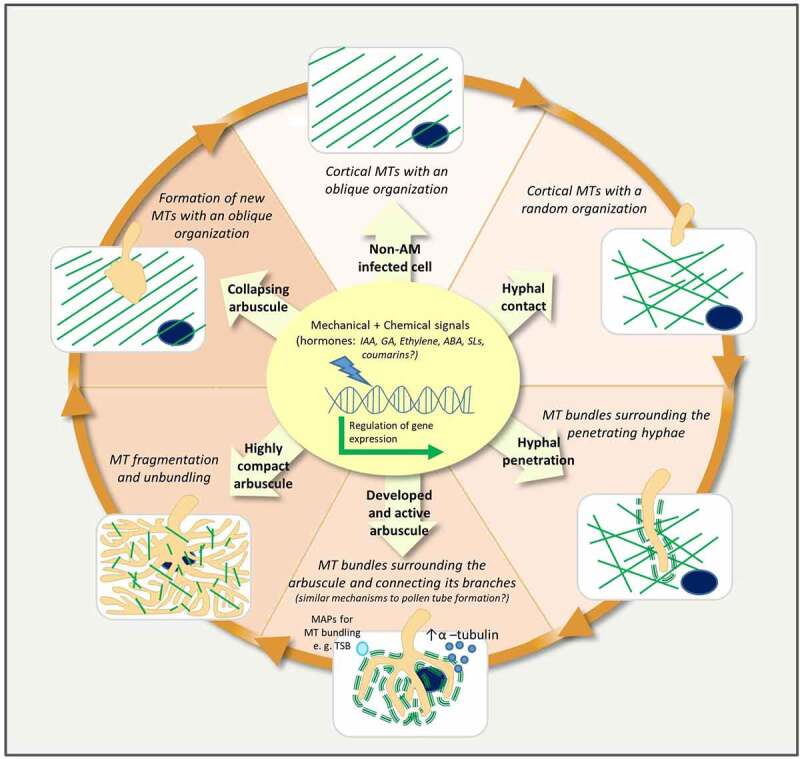
Model of microtubule modifications in the root hosting cell along different stages of the arbuscule life cycle, and the underlying signals and mechanisms involved. A hypothetical model is illustrated, in which the mechanical stimulus caused by the penetrating fungus combined with different hormone balances in the plant cell, trigger a differential regulation of gene expression along different stages, consequently determining the action of specific mechanisms for the continuous modifications of the MT cytoskeleton. During arbuscule development, MT rearrangements are essential for arbuscule functionality, and the mechanisms involved might be common to those ones occurring in pollen tube formation, with the participation of specific MAPs for MT bundling, such us the TSB in tomato.

Huge microtubule changes in cells colonized by AM fungi have been widely reported in tobacco and Medicago,^3,5^ with a particular interest on the arbuscule-containing cells along the different stages of arbuscule formation, as shown in Figure 1. However, up to date, little is known about the triggering signals, the underlying mechanisms and the role of such MT modifications. Only a recent study has identified the importance of MT bundling for arbuscule formation and functionality in tomato through the functional analysis of the *Tsb* gene, encoding a putative MAP.^1^ The identification of *Tsb* functional orthologous in other species may help to validate if this mechanism is conserved in all mycorrhizal species. With this purpose, given the possible similarities between the MT cytoskeleton changes occurring in the pollen tube development and in the AM colonized roots, an interesting approach would be to pinpoint genes encoding MAPs, upregulated in both conditions or containing both pollen- and AM-related predicted regulatory sequences in their promoters. In addition, it is important to consider that MT rearrangements also occur and should have a role at later stages of arbuscule development, during arbuscule senescence and collapse. In this sense, further approaches based on live imaging and biochemical techniques joined to comparative transcriptomic analysis on microdissected arbusculated cells at different stages of arbuscule development may help to identify MAPs and other players involved, and to elucidate the role of the MT modifications occurring at each specific stage of the arbuscule life cycle.
